# Detection of Impaired Cognitive Function in Rat with Hepatosteatosis Model and Improving Effect of GLP-1 Analogs (Exenatide) on Cognitive Function in Hepatosteatosis

**DOI:** 10.1155/2014/946265

**Published:** 2014-03-11

**Authors:** Oytun Erbaş, Fulden Sarac, Hüseyin Aktuğ, Gönül Peker

**Affiliations:** ^1^Department of Physiology, Faculty of Medicine, Gaziosmanpasa University, Tokat, Turkey; ^2^Department of Internal Medicine, School of Medicine, Ege University, Izmir, Turkey; ^3^Department of Geriatrics Medicine, Medical Faculty, Ege University, 5th Floor, Bornova, 35100 Izmir, Turkey; ^4^Department of Histology and Embryology, School of Medicine, Ege University, Izmir, Turkey; ^5^Department of Physiology, School of Medicine, Ege University, Izmir, Turkey

## Abstract

The aims of the study were to evaluate (1) detection of cognitive function changing in rat with hepatosteatosis model and (2) evaluate the effect of GLP-1 analog (exenatide) on cognitive function in hepatosteatosis. In the study group, 30% fructose was given in nutrition water to perform hepatosteatosis for 8 weeks to 18 male rats. Six male rats were chosen as control group and had normal nutrition. Fructose nutrition group were stratified into 3 groups. In first group (*n* = 6), intracerebroventricular (ICV) infusion of exenatide (*n* = 6) was given. ICV infusion of NaCl (*n* = 6) was given to second group. And also, the third group had no treatment. And also, rats were evaluated for passive avoidance learning (PAL) and liver histopathology. Mean levels of latency time were statistically significantly decreased in rats with hepatosteatosis than those of normal rats (*P* < 0.00001). However, mean level of latency time in rats with hepatosteatosis treated with ICV exenatide was statistically significantly increased than that of rats treated with ICV NaCl (*P* < 0.001). Memory performance falls off in rats with hepatosteatosis feeding on fructose (decreased latency time). However, GLP-1 ameliorates cognitive functions (increased latency time) in rats with hepatosteatosis and releated metabolic syndrome.

## 1. Introduction

The metabolic syndrome (MetS), a disease arising from the worldwide epidemic of obesity, is manifested as severe insulin resistance, hyperlipidaemia, hepatic steatosis, and diabetes [1]. And also MetS has been shown to predispose cognitive impairment [2–4]. Recent studies addressing the association of MetS with cognitive performance and risk for dementia report mixed results [5–7]. Potential explanatory models include impaired vascular reactivity, neuroinflammation, oxidative stress, and abnormal brain lipid metabolism [8]. In a study, Su et al. [9] suggested that rats with the metabolic syndrome have ineffective inflammation-resolving mechanisms that represent plausible reasons for the exaggerated and persistent postoperative cognitive decline. And also inflammation induces deficits in neurotransmitter-releasing neurons such as cholinergic, noradrenergic, and serotonergic neurons [10–12]. In previous studies [13–15], glucagon-like peptide-1 (GLP-1) and its receptor (GLP-1R) modulated neuronal activity and protected against neuronal damage induced by various insults in the brain. And they were involved in learning and memory. However, there were not many studies related to investigating the showing impaired cognitive function in rat with hepatosteatosis and GLP-1 effect on cognitive function. Therefore, the aims of the study were to evaluate (1) detection of cognitive function changing in rat with hepatosteatosis model and (2) evaluate the effect of GLP-1 analog (exenatide) on cognitive function in hepatosteatosis.

## 2. Materials and Methods

### 2.1. Animals

In this study 24 male Sprague Dawley albino mature rats at 8 weeks, weighing 200–220 g, were used. Animals were fed ad libitum and housed in pairs in steel cages having a temperature-controlled environment (22 ± 2°C) with 12 h light/dark cycles. The experimental procedures were approved by the Committee for Animal Research of Ege University. All animal studies are strictly conformed to the animal experiment guidelines of the Committee for Human Care.

### 2.2. Experimental Protocol

In the study group, 30% fructose was given in nutrition water to perform hepatosteatosis for 8 weeks to 18 male rats. Six male rats were chosen as control group and had normal nutrition.

**Figure 1:**
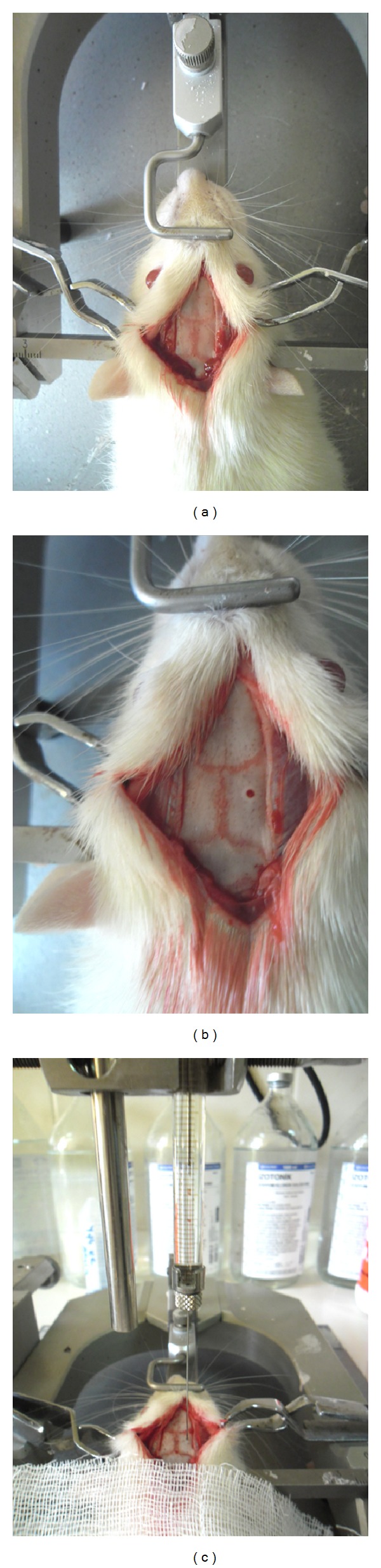
Stereotaxical infusion protocol was shown in rat.

Fructose nutrition group were stratified into 3 groups. In the first group (*n* = 6), intracerebroventricular (ICV) infusion of exenatide (*n* = 6) was given. ICV infusion of NaCl (*n* = 6) was given to the second group. And also the third group had no treatment. ICV injection was performed under anesthetized. Rats were deeply anesthetized by the mixture of ketamine hydrochloride (40 mg/kg, Alfamine, Ege Vet, Alfasan International B.V., Holland) and xylazine hydrochloric (4 mg/kg, Alfazyne, Ege Vet, Alfasan International B.V., Holland), i.p., and placed in a stereotaxic frame (Figure 1). Exenatide 10 *μ*g/kg (Byetta, Lilly) was infused 10 *μ*L into the left lateral ventricle (AP = −0.8 mm; ML = ±1.6 mm; DV = −4.2 mm) with a 28-gauge Hamilton syringe in. Sham-operated rats received vehicle (10 *μ*L isotonic NaCl). The needle was left in place for an additional 2 min for complete diffusion of the drug. All animals were given penicillin intraperitoneally to prevent postsurgical infection. After surgery, rats were weighed regularly and monitored daily for behavior and health conditions.

After 5-day recovery phase, passive avoidance task evaluating the memory was performed to study and control groups. This passive avoidance learning (PAL) is a one trial fear-motivated avoidance task in which the rat learns to refrain from stepping through a door to an apparently safer but previously punished dark compartment. The PAL box which is equal to the size of dark and light sections used. This box has a grid system which performs electric shock in dark compartment. Normally, rats when placed in the light compartment prefer to enter dark compartment. After 10 sec of habituation, the guillotine door separating the light and dark chambers was opened. When the rat passed to dark compartment, door between the light and dark compartments was closed. Then, 1.5 mA electric shock was applied for 3 seconds and the rat was removed from the dark chamber and returned to its home cage. After 24 hours, rats were placed in the same mechanism. The time (latency) to switch from light compartment to dark compartment of rats was recorded but foot shock was not delivered, and the latency time was recorded up to a maximum of 300 sec. The latency to refrain from crossing into the punished compartment serves as an index of the ability to avoid, and allows memory to be assessed. Then, the animals were euthanized and hepatectomy was performed for histopathological examination.

### 2.3. Histopathological Examination of Liver

For histological and immunohistochemical studies, all animals were anesthetized by an i.p. of ketamine (40 mg/kg)/xylazine (4 mg/kg) and perfused with 200 mL of 4% formaldehyde in 0.1 M phosphate-buffer saline (PBS). Formalin-fixed liver sections (4 *μ*m) were stained with hematoxylin-eosin (H&E). All sections were photographed with Olympus C-5050 digital camera mounted on Olympus BX51 microscope.

Morphological analysis was assessed by computerized image analysis system (Image-Pro Express 1.4.5, Media Cybernetics, Inc., USA) on ten microscopic fields per section examined at a magnification of ×40, with the observer blind to the study group. The liver pathologic findings were scored as follows [16] steatosis (the percentage of liver cells containing fat). The total liver pathology score was calculated by adding the scores from each parameter:

 1+, less than 25% of cells containing fat;
 2+, 26% to 50%;
 3 + 51% to 75%; and
 4+, more than 75%;
 inflammation and necrosis: one focus/lobul 1+;
 Two or more foci/lobule 2+.

### 2.4. Statistical Analysis

Statistical evaluation was performed using SPSS version 15.0 for Windows. The groups of parametric variables were compared using the Student's *t*-test and analysis of variance. Also, the groups of nonparametric variables were compared using the Mann-Whitney *U* test. In addition, the Shapiro-Wilk test was used for parametric-nonparametric differentiation. Results are presented as mean + SEM. A *P* < 0.05 was accepted as statistically significant.

## 3. Results 

**Figure 2:**
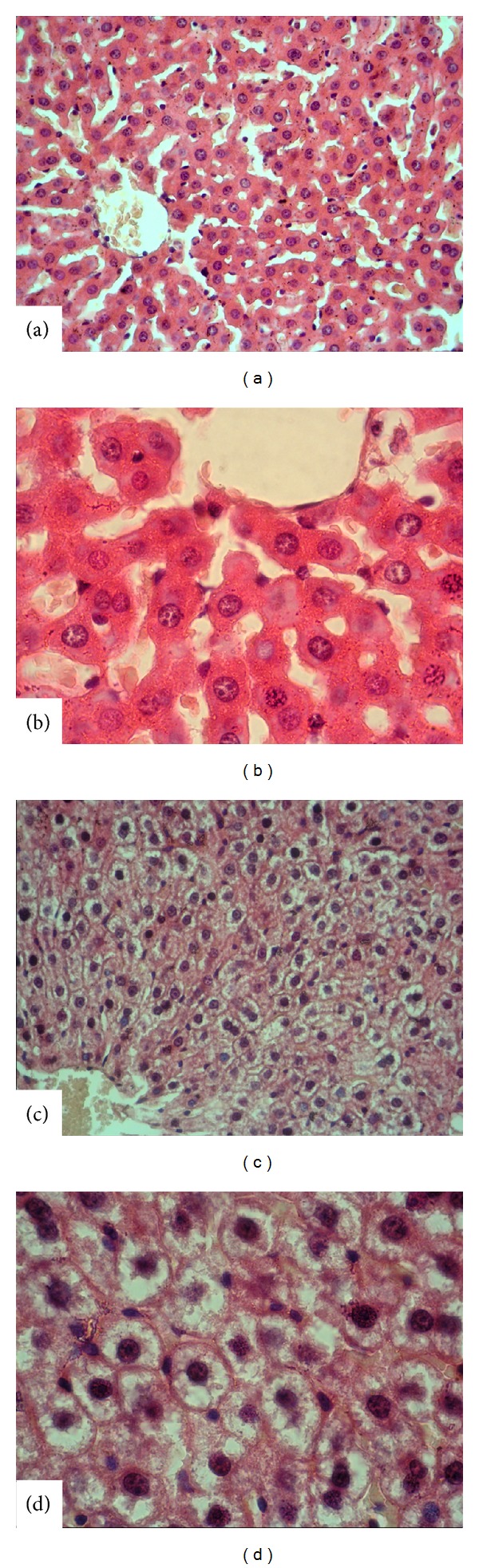
Liver histopathology ((a)-(b)): normal group liver, H&E (×40 and ×100 magnification), and ((c)-(d)): hepatosteatosis group liver (macrovesicular steatosis in liver cytoplasm).

Macrovesicular hepatosteatosis was shown in rat who had received fructose. It was found that latency time was statistically significantly decreased in rat with hepatosteatosis (*P* < 0.00001) (Figure 2).

Mean levels of latency time in rats with hepatosteatosis treated with ICV GLP-1 analog (exenatide) was statistically significantly increased (memory improvement) than those of rats with hepatosteatosis treated with ICV isotonic NaCl (*P* < 0.001) (Table 1).

Mean levels of histopatologic score of liver were not significantly statistically different in rat with hepatosteatosis treated with ICV NaCl or ICV exenatide or anything (*P* > 0.05).

Mean levels of latency time were statistically significantly decreased in rats with hepatosteatosis than those of normal rats (*P* < 0.00001). However, mean level of latency time in rats with hepatosteatosis treated with ICV exenatide was statistically significantly increased than that of rats with hepatosteatosis treated with ICV NaCl (*P* < 0.001).

## 4. Discussion

Overconsumption of sugar-sweetened beverages promotes the development of overweight and is associated with metabolic disturbances, including intrahepatic fat accumulation and metabolic syndrome [17]. In the present study, macrovesicular hepatosteatosis was found in rat that received fructose overconsumption. And also it was found that cognitive performance (decreased latency time) has been impaired in rat with hepatosteatosis compared to normal rat. However, cognitive functions were improved with GLP-1 analog such as exenatide treatment.

Diet can affect brain plasticity and cognitive functions such as learning and memory [18]. A high fructose diet produces large increases in plasma triglyceride concentrations and liver mass [19]. In addition to increases in visceral adipose tissue [20–22] and liver mass [23, 24] with high fructose diet in both male and female rats, behavioral status can change. In previous studies [25–27], acute injections of fructose have affected the cognitive functions in animals. Likewise, Ross et al. [28] reported that a high fructose diet impaired spatial water maze memory in male rats. Likewise, in the present study, mean levels of latency time were statistically significantly decreased in rats with hepatosteatosis than those of normal rats. However, Bruggeman et al. [29] suggested that the metabolism of fructose and the effects of a high fructose diet on learning and memory might be sex dependent.

GLP-1, an endogenous 30-amino-acid peptide produced in enteroendocrine cells of intestine, stimulates glucose-dependent insulin secretion [30, 31]. And also GLP-1 and GLP-1R have been considered a therapeutic target in neurodegenerative and cognitive disorders throughout the central and peripheral nervous systems [14, 15, 32, 33]. In a previous study, Oka et al. [14] indicated that GLP-1 receptors existed in the hippocampus and are involved in modulating hippocampal activity through an increase in the release of excitatory amino acid transmitters. Likewise, Perry et al. [32] reported that GLP-1R has been considered a therapeutic target in neurodegenerative and cognitive disorders throughout the central and peripheral nervous systems. And also GLP-1 has been reported to cross the blood-brain barrier (BBB) and facilitate insulin signaling [34, 35]; exenatide, a GLP-1 receptor agonist, could enhance neuronal progenitor proliferation in the brain of diabetic mouse [35]. Recently, Chen et al. [36] suggested that GLP-1 receptor agonist can protect neurons from diabetes-associated glucose metabolic dysregulation insults in vitro and from ICV streptozotocin insult in vivo. In another study, Gault et al. [37] showed that GLP-1 receptor agonist therapy improved cognitive function and ameliorated impaired hippocampal synaptic plasticity in dietary-induced obesity. In the present study, we found that mean levels of latency time in rats treated with ICV exenatide were increased than those of rats treated with ICV NaCl. To our findings, exenatide treatment improved learning and memory performance in rats with hepatosteatosis and metabolic syndrome. So, it may be a candidate for alleviation of memory and cognitive dysfunctions in metabolic disorders.

In summary, memory performance falls off in rats with hepatosteatosis feeding with fructose (decreased latency time). However, GLP-1 ameliorates cognitive functions (increased latency time) in rats with hepatosteatosis and related metabolic syndrome.

## Figures and Tables

**Table 1:** Mean levels of latency time and histopathologic score were shown in study and control groups.

	Normal	Rat with hepatosteatosis (no treatment)	Rat with hepatosteatosis and ICV NaCl	Rat with hepatosteatosis and ICV exenatide
Latency time (sec)	161.5 ± 14.9	16.3 ± 1.8*	14.1 ± 0.9*	73.8 ± 6.4^#^
Histopathologic score	1.14 ± 0.12	5.38 ± 0.35**	5.41 ± 0.52	5.26 ± 0.48

Results were presented as mean ± SEM. *P < 0.00001, **P < 0.05 different from normal groups; ^#^ *P* < 0.001 different from rat with hepatosteatosis (no treatment) and rat with hepatosteatosis given ICV NaCl.
